# Unveiling the Distinct Effects of a Two-Dimensional Copper/Sodium Complex: Oxidative Stress on Erythrocytes and Cytotoxicity, Apoptosis, Drug Resistance, and Inflammation in Lung Cancer Cells

**DOI:** 10.2174/0118715206380073250811113008

**Published:** 2026-09-03

**Authors:** Chenchen Li, Mostafa Heidari Majd, Ameneh Heidari, Zohreh Razmara, Dongdong Guo

**Affiliations:** 1 Department of Thoracic Cancer Surgery, Anhui Provincial Cancer Hospital, Hefei, 230031, China;; 2 Department of Medicinal Chemistry, Faculty of Pharmacy, Zabol University of Medical Sciences, Zabol, 9861615881, Iran;; 3 Department of Chemistry, Faculty of Science, University of Zabol, Zabol, 9861335856, Iran;; 4 Department of Medical Oncology, Yan'an People's Hospital, Yan'an, 716000, China

**Keywords:** Apoptosis, Bak1, TNF-α, metal complex, AKT1, CTR1

## Abstract

**Introduction:**

Copper complexes, as endogenous metals, have potential in cancer therapy, addressing issues associated with cisplatin. Since cisplatin uses Copper Transporter 1 (CTR1) for cellular entry, copper complexes may utilize this pathway to enhance transport efficiency.

**Methods:**

The Cu/Na dipicolinic acid complex was synthesized to assess its cytotoxicity, induction of apoptosis, drug resistance, and inflammation in cancerous and normal lung cells. The effects of oxidative stress on erythrocytes were also examined.

**Results:**

Cytotoxicity tests (MTT and SRB) showed superior inhibitory effects on A549 lung cancer cells compared to cisplatin, with no toxicity observed in MRC-5 normal lung fibroblast cells. Real-time PCR revealed increased caspase-3 expression (extrinsic apoptosis) for the complex compared to cisplatin, possibly due to CTR1-mediated entry. The complex did not induce drug resistance, as shown by AKT1 expression, and reduced TNF-α expression, preventing inflammation in normal cells. In contrast to cisplatin, the complex caused minimal oxidative stress in erythrocytes.

**Discussion:**

It can be concluded that the Cu/Na dipicolinic acid complex may be easily transported by CTR1 to malignant tumors, particularly lung cancer. This complex has the ability to inhibit cancer cell growth and induce apoptosis in lung cancer cells. Therefore, copper complexes show promise as potential therapeutic options for treating this type of cancer.

**Conclusion:**

The copper/sodium complex demonstrates enhanced therapeutic efficacy in lung cancer cells, requiring lower doses than cisplatin, while being safer for normal cells and erythrocytes.

## 1 INTRODUCTION

Several studies have shown that copper metal ions activate Superoxide Dismutase (SOD), an endogenous antioxidant. It has also been confirmed that a copper complex with GHK, a tripeptide made up of glycyl-histidyl-lysine, can decrease the production of Reactive Oxygen Species (ROS), boost SOD activity, and lower the release of pro-inflammatory cytokines in cases of acute lung injury [1, 2]. Furthermore, the GHK-Cu complex in lung tissue affected by pulmonary fibrosis has been found to reduce the inflammatory response by decreasing the levels of inflammatory cytokines, such as TNF-ɑ and IL-6 [3].

Copper complexes are excellent candidates for biological applications due to their high bioavailability, wide structural diversity, positive redox potential, modulation of intracellular Reactive Oxygen Species (ROS) levels, and low toxicity to normal cells [4]. Compared to non-endogenous metals like platinum, copper complexes, as an endogenous metal, can be valuable tools for cancer treatment and may help eliminate the issues associated with the approved drug cisplatin [5]. It has been proven that platinum complexes, such as cisplatin, enter cancer cells more effectively through the Copper Transporter 1 (CTR1) and exert their therapeutic effect within these cells [6]. Given that copper is transported into cancer cells by the CTR1 transporter more effectively than platinum [7], it can be expected that the primary route of entry for copper complexes is also through CTR1. This suggests that copper complexes can enter cancer cells more easily and exhibit stronger anticancer effects. Research has shown that once copper complexes enter cancer cells, they can induce apoptosis by activating stress pathways, causing cell cycle arrest, inhibiting angiogenesis, promoting coproptosis and paraptosis [8]. As a result, copper complexes have become significant candidates for cancer treatment.

The most important aspect of copper complexes is their ability to employ different mechanisms of action in treating cancer cells, potentially reducing drug resistance. Unlike traditional chemotherapeutic agents like cisplatin, copper complexes can: induce oxidative stress, causing damage to cancer cell components like DNA, proteins, and lipids, ultimately leading to cell death; disrupt mitochondrial function, thereby disrupting the energy supply of cancer cells; inhibit the formation of new blood vessels that tumors require for growth and survival; trigger programmed cell death in cancer cells through specific signaling pathways; and bind to DNA and interfere with its replication and transcription, both crucial processes for cancer cell proliferation. These diverse mechanisms of action reduce the likelihood of cancer cell resistance as they target multiple pathways simultaneously. This versatility makes copper complexes a promising area for research in oncology. Given the high drug resistance seen with cisplatin in cancer treatment, the advantages of copper complexes are particularly promising [9]. Various copper complexes have been synthesized by binding with different ligands, and their effectiveness has been studied in the treatment of various types of cancers. In the meantime, pyridine-2,6-dicarboxylic acid (dipicolinic acid) has been shown to possess strong N-O chelation properties. This enables it to effectively bind to a copper ion by utilizing the oxygen atoms in the carboxylate groups and the nitrogen atom in the pyridine ring, allowing for the synthesis of a water-soluble complex [10].

In terms of metabolism and prevention of excessive copper accumulation in the body, there is a certain advantage over cisplatin. Due to the high redox activity of copper, in the physiological environment of the body, the amount of copper is strictly controlled in case of possible release from the ligand. Copper is usually not present as a free ion but is bound to a specific protein in the liver. The liver plays a crucial role in copper deposition, with most of the accumulated copper being bound to Metallothionein (MT), a low molecular-weight, metal-rich, cysteine-rich protein [11]. MT has a high affinity for metals and protects against metal toxicity, entering the intestine with hepatic bile. This allows the copper needed for essential body processes to be reabsorbed through intestinal epithelial cells and enter the bloodstream, while any excess is ultimately excreted, maintaining intracellular copper levels below toxic levels [12].

Considering all the mentioned facts and the importance of copper as a vital element for the proper functioning of enzyme pathways, particularly those involving cytochrome oxidase and mitochondrial respiration [13], a copper complex was resynthesized using dipicolinic acid as a ligand. Then, its cytotoxic, apoptotic, and drug resistance effects on the A549 lung cancer cell line were evaluated. Additionally, the cytotoxic and inflammatory effects of the complex were examined on MRC-5 fetal lung fibroblast cells to determine any functional distinctions when compared to cisplatin.

For this study, the cytotoxic effects were investigated using the MTT method and Sulforhodamine B (SRB) staining method on both cell lines. Additionally, the expression levels of genes involved in the mitochondrial pathway of apoptosis, such as Bclx and Bak1, and the external pathway of apoptosis, such as Caspase-3, in the A549 cell line were analyzed using real-time PCR. Furthermore, the level of AKT1 gene expression in cancer cell lines was assessed to identify potential resistance to treatment and metastasis. Lastly, the study examined expression levels of genes involved in inflammatory responses, such as TNF-α, to evaluate any potential side effects of the copper complex and ensure it does not pose a risk to normal lung cells. For comparison, all tests are also performed on cisplatin, a known pharmaceutical complex.

## 2 MATERIALS AND METHODS

### 2.1 Materials

Pyridine-2,6-dicarboxylic acid (dipicolinic acid), Phosphate-Buffered Saline (PBS), Sulforhodamine B (SRB), and nitric acid (HNO_3_) were provided by Sigma-Aldrich in Taufkirchen, Germany. Sodium hydroxide (NaOH), potassium bromide (KBr), MTT (3-(4,5-dimethylthiazol-2yl)-2,5 diphenyl tetrazolium bromide), copper(II) nitrate trihydrate (Cu(NO_3_)_2_·3H_2_O), trichloroacetic acid (TCA), thiobarbituric acid (TBA), triethanolamine (TEA), and Tris base were obtained from Merck in Darmstadt, Germany. RPMI was sourced from VIVA CELL in Isfahan, Iran. Fetal Bovine Serum (FBS) was purchased from PAN Biotech in Aidenbach, Germany. Penicillin/streptomycin and trypsin were acquired from Gibco in Carlsbad, USA. A549 lung cancer cells and MRC-5 fetal lung fibroblast cells were obtained from the Pasteur Institute in Tehran, Iran.

### 2.2 Resynthesis of a Two-Dimensional Multi-Aqueous Cu/Na Dipicolinic Acid Complex

To prepare the Cu/Na dipicolinic acid complex, [Cu(μ-dipic)_2_{Na_2_(μ-H_2_O)_4_}]_n_·2nH_2_O, a method outlined in two published articles was followed [10, 14]. First, 1 mmol of Cu(NO_3_)_2_·3H_2_O (241.6 mg) was mixed with 1 mmol of HNO_3_ (63.01 mg) to prevent the spontaneous hydrolysis of the metal salt, resulting in a 10 mL solution. An ultrasonic probe was used to stir and homogenize the solution throughout the reaction. Subsequently, 130 µL of TEA (1 mmol) was added dropwise to the solution to create an alkaline environment. Then, 160 mg of NaOH (4.00 mmol) was added to deprotonate the solution. Finally, 167.12 mg of dipicolinic acid (1 mmol) was added, and the mixture was stirred for two hours using an ultrasonic probe at room temperature. The resulting blue solution was then reduced in volume using a rotary evaporator under vacuum. The final 2 mL were dried completely by exposure to the environment. To identify the final product, its FTIR spectrum was obtained using a Shimadzu device after preparing KBr tablets. Additionally, our previously published work [10], reported complementary studies on this compound, including the identification of its structure, DNA binding constant, stability, Hirshfeld surface analysis, viscosity, partition coefficients, fluorescence spectral data, docking method, and other related analyses.

### 2.3 Investigation of Cytotoxic Effects by the MTT Method

This procedure is based on the color change of yellow MTT to purple formazan, which occurs due to the function of the mitochondrial enzyme succinate dehydrogenase in living cells. Two cell lines, A549 (human lung adenocarcinoma) and MRC-5 (human fetal lung fibroblasts), were obtained from the Pasteur Institute (Tehran, Iran) to investigate the cytotoxic effects of Cu/Na dipicolinic acid complex compared to cisplatin. To begin, the two cell lines were cultured in RPMI-1640 growth medium with 10% FBS and 1% penicillin G-streptomycin for three weeks until reaching the desired number [15]. Once the flasks reached 80% confluence, cells were seeded into 96-well plates at a density of 1.5 × 10^4^ cells per well and allowed to adhere for 24 hours in a CO_2_ incubator under standard conditions. Five different concentrations of Cu/Na dipicolinic acid complex (ranging from 6.25 µM to 100 µM) were prepared in the growth medium to treat the A549 cells for 48 and 72 hours. For the MRC-5 normal cell line, the concentrations were similar to those used for the cancer cell line; however, the exposure time was only 72 hours. Each concentration was repeated three times independently (n = 3, ± SD) to minimize error. Additionally, three wells served as negative controls for each time period. To compare, five similar concentrations of the common anticancer drug cisplatin were added to the cells as a positive control under the same conditions. After the appropriate incubation times, the medium in each well was carefully replaced with 150 µL RPMI-1640 and 50 µL of MTT solution (5 mg/mL in PBS), then incubated for an additional 4 hours in the dark at 37 °C. Subsequently, the medium was removed and the resulting formazan crystals were solubilized using 200 µL of DMSO per well. The samples were then prepared for Optical Density (OD) measurements at 570 nm using an ELISA reader (Biochrom Anthos 2020 Microplate Reader, London, UK).

### 2.4 Investigation of Cytotoxic Effects via the SRB Method

This procedure utilizes cellular protein content to assess cytotoxicity. The methodology involves staining cellular proteins with SRB dye, resulting in a quantifiable colorimetric change [16]. A549 lung cancer cells were used for this test, as mentioned in the previous MTT assay. The cells were cultured in RPMI-1640 medium supplemented with 10% FBS and 1% penicillin-streptomycin. Once the cells had reached sufficient growth, they were seeded into 96-well plates at a density of 1.5 × 10^4^ cells per well. Various concentrations of the Cu/Na dipicolinic acid complex and cisplatin (ranging from 6.25 µM to 100 µM) were then administered. The A549 cells were exposed to these compounds for 48 and 72 hours. After removing the culture medium containing drugs, 100 µL of cold 10% TCA was added to each well, and the plate was stored at 4°C in the dark for 60 minutes to allow protein precipitation. The excess TCA in the wells was then washed away with sterile distilled water, and the plate was gently dried with air. In the next step, 100 µL of 0.5% SRB solution was added to each well and incubated again for 30 minutes in the dark at room temperature. Residual SRB was removed using a 0.5% acetic acid solution, followed by gentle air-drying. Finally, 200 µL of Tris base (10 mM) was added to each well and gently shaken for 20 minutes to solubilize the dye. The absorbance of the pink-stained cells was read at 510 nm using an ELISA reader (Biochrom Anthos 2020 Microplate Reader, London, UK).

### 2.5 Analysis of Gene Expression via Real-Time PCR

This procedure examines the expression of specific genes involved in apoptosis and inflammation in two distinct cell lines using RNA isolation, cDNA synthesis, and real-time PCR analysis. A549 lung cancer cells were studied to examine the expression of Bak1, Bclx, Caspase-3, and AKT1 genes. MRC-5 fetal lung fibroblast cells were studied to investigate genes involved in inflammation, specifically TNF-α. Three separate flasks of each cell line (three flasks of A549 and three flasks of MRC-5) containing two million cells were prepared. In three A549 flasks: Flask 1 contained 6 µM Cu/Na dipicolinic acid complex, Flask 2 contained 10 µM cisplatin, and Flask 3 contained empty culture medium (control). In three MRC-5 flasks: Flask 1 contained 247 µM Cu/Na dipicolinic acid complex, Flask 2 contained 5 µM cisplatin, and Flask 3 contained empty culture medium (control). The concentrations were selected based on IC_50_ values, and the cells were incubated for 24 hours. To begin the RNA isolation protocol, the culture medium was removed from the flasks, and the cells were washed twice with PBS. The cells were then detached from the bottom of the flasks using trypsin and collected by centrifugation in six separate Falcon tubes. The RNA isolation protocol was carried out using the SinaPureTM RNA kit (SinaClon, Tehran, Iran) as follows:

(i) Lysis solution was added to each Falcon and vortexed. (ii) The precipitation solution was added, and the Falcons were shaken ten times. (iii) The solutions were transferred to tubes with collector columns and incubated with DNase solution. (iv) After centrifugation, the bottom solutions were discarded. (v) The washing solution I was added, centrifuged, and the bottom solutions were discarded. This step was repeated. (vi) Washing solution II was added, centrifuged, and the bottom solutions were discarded. (vii) RNase-free water was added, and the RNA was eluted through centrifugation.

The SambioTM cDNA Synthesis Kit (Tehran, Iran) was used for cDNA synthesis. 2 µL of SamScript Enzyme, 10 µL of 2x Reaction Buffer, and 8 µL of isolated RNA were combined and then incubated for 10 minutes at 25°C, 60 minutes at 47°C, and 5 minutes at 85°C. Six cDNA samples were synthesized.

Gene expression analysis was performed using the Evagreen qPCR dye, with GAPDH as a housekeeping gene for normalization. The fold change in gene expression was calculated using the ΔΔCT method. The expression of Bclx, Bak1, AKT1, and Caspase-3 genes from cancer cells, as well as the TNF-α gene from normal cells (Table **1**), was evaluated using real-time PCR on the Applied Biosystems StepOne™ system (Thermo Fisher Scientific, Massachusetts, USA) [17].

### 2.6 Determining Oxidative Damage in Erythrocytes

Approximately 200 mL of heparinized blood was obtained from the Zabol Blood Transfusion Organization. The blood was then centrifuged for half an hour (2 ×) at 3000 rpm to separate pure erythrocytes [18]. In the next step, the pure erythrocytes were washed (2 ×) with PBS, and a 1:9 mixture of red blood cells and PBS was prepared. Using this mixture, two concentrations (6 and 12 µM) of both the Cu/Na dipicolinic acid complex and cisplatin were prepared with a final volume of 5 mL. The mixtures were then incubated at 37°C for 24 hours. After 24 hours, 1 mL of each sample was removed and added to a 2:1 mixture of TCA (20%) and TBA (0.028 M). The samples were then incubated at 100°C for 15 minutes. The supernatant was centrifuged, and the absorbance was measured at 532 nm to determine the amount of Malondialdehyde (MDA) produced by the erythrocytes. All steps were repeated three times (n = 3) to eliminate possible errors.

## 3 RESULTS AND DISCUSSION

### 3.1 Examining the Characteristics of the Cu/Na Dipicolinic Acid Complex

Dipicolinic acid has a strong ability to form complexes with metal ions, protecting them from degradation and maintaining the integrity and stability of metal complexes [19]. In the past, it has been used to enhance the solubility of drugs. When bound to metals, it creates water-soluble complexes that exhibit good bioavailability and can easily traverse biological membranes [20]. Previous studies have shown that the Cu/Na dipicolinic acid complex (Fig. **1**) dissolves at a concentration of 15 mg/mL in water (at a temperature of 25°C) and displays excellent stability in aqueous media [14]. Since the compound has already been identified using various methods such as UV-VIS, mass spectrometry, and FTIR in previous articles [10], we decided to prepare the FTIR spectrum and compare it with the published data to ensure the accuracy of the synthesis.

As shown in Fig. (**2**) of the FTIR spectrum, the broad multi-peak in the range of 3400-3500 cm^-1^ represents the presence of H_2_O molecules in the complex structure. Additionally, the peak associated with asymmetric COO is observed at around 1623 cm^-1^, and the peak for symmetric COO is found in the region of 1383 cm^-1^. To confirm the presence of pyridine in the complex structure, one can examine the peak at 3067 cm^-1^, which corresponds to the aromatic C-H bonds.

### 3.2 Cytotoxicity Test on Cancer and Normal Cells

In humans, copper is an essential metal element in the body and a crucial cofactor for various biological processes [21]. Copper binds to enzymes to facilitate blood clotting, hormonal maturation, and cellular energy processing [22]. Interestingly, the growth and metastasis of tumors are heavily reliant on copper. By stimulating angiogenesis, copper plays an important role in activating and supporting tumorigenesis through the phosphoinositide 3‐kinase (PI3K)‐Protein Kinase B (PKB, also termed AKT) oncogenic signaling pathway [23]. Excessive angiogenesis can be associated with elevated copper levels, which may be readily transported by the copper transporter 1 and support the development of malignant tumors. Cancer patients not only show high levels of copper in tumors, but also exhibit higher serum copper levels in general [24]. However, the involvement of copper in tumorigenesis is a double-edged sword. The Cu/Na dipicolinic acid complex, if copper ions are released, could theoretically contribute to cancer properties through interactions with cellular pathways. However, it should be noted that copper is converted into a stable complex, and the cytotoxic effects of the Cu/Na dipicolinic acid complex should be carefully evaluated. While copper ions released from this complex may theoretically activate angiogenesis and PI3K/AKT signaling, the specific structural features and targeted delivery of the complex may limit the availability of its free copper ion, thus reducing the potential activation of oncogenic pathways.

Therefore, the use of copper in metal complexes can help facilitate entry into tumors, particularly in cases of lung cancer. Copper accumulation in the lungs has been proven [25], making copper complexes potential therapeutic options for this type of cancer. Due to the formation of the complex, the ionic nature of copper is altered, and the ligands exhibit the ability to treat cancer. Researchers have developed various copper complexes that have shown promising results in the treatment of tumors [5]. Numerous studies have been conducted on the induction of autophagy and apoptosis in tumor cells by copper and its complexes [26]. Accordingly, various mechanisms have been uncovered, including the activation of stress pathways, cell cycle arrest, inhibition of angiogenesis, cuproptosis, and paraptosis [8]. One of the special ligands is dipicolinic acid, which easily coordinates with copper and has shown effectiveness in cancer treatment [27]. Therefore, we decided to investigate the effect of Cu/Na dipicolinic acid complex on the A549 lung cancer cell line.

As shown in Fig. (**3A**), the Cu/Na dipicolinic acid complex was able to inhibit the growth of A549 lung cancer cells. Its performance is almost as good as cisplatin and inhibits growth more than cisplatin at all concentrations. Over time, this effect has increased, leading to the conclusion that the complex inhibits the growth of cancer cells in a time and concentration-dependent manner. An interesting observation is the effectiveness of the lowest concentration (6.25 µM) after 72 hours of exposure to cancer cells. Cisplatin was unable to reduce the percentage of cell viability to 50%, but the complex was successful in achieving this goal.

The superiority of the complex in inhibiting the growth of lung cancer cells compared to cisplatin can also be understood from the IC_50_ values (Fig. **3B**). At both 48 hours and 72 hours, the IC_50_ value of the Cu/Na dipicolinic acid complex is lower compared to cisplatin.

The conversion of tetrazolium salt MTT to water-insoluble purple formazan crystal is carried out in living cells *via* mitochondrial succinate dehydrogenase [28]. Various metabolic factors, such as mitochondrial and non-mitochondrial oxidoreductase activity, oxidative stress, glucose levels, lactate levels, and cellular metabolic disorders, may significantly limit the use of the MTT method [29]. Therefore, it is necessary to employ another method to ensure the effectiveness of medicinal compounds against cancer cells. As a result, we utilized the method of measuring protein content in cells using SRB staining to confirm the impact of the complex on cancer cells. In this method, SRB dye is stoichiometrically bound to proteins in mildly acidic conditions to determine the amount of cell mass from the amount of dye and evaluate the extent of cell proliferation [16].

Once again, the results from Fig. (**4A**), obtained using the SRB staining method, confirmed that the Cu/Na dipicolinic acid complex can effectively inhibit the growth of A549 lung cancer cells and outperforms the well-known drug cisplatin at both 48 and 72 hours. This is supported by the IC_50_ values derived from the curve plotting the logarithm of the concentration against the percentage of cell viability (Fig. **4B**). In theory, the IC_50_ values obtained through the SRB method are typically higher than those obtained through the MTT method, indicating poorer performance. This discrepancy may be attributed to the low protein content within living cells [30].

In Fig. (**1**), it is evident that the size of the Cu/Na dipicolinic acid complex is larger compared to cisplatin due to having a bulky ligand. However, it can be assumed that the presence of copper in the structure of the Cu/Na dipicolinic acid complex facilitates its entry into lung cancer cells more effectively *via* CTR1 [31], thereby increasing its effectiveness. Of course, CTR1 is also a transporter of platinum drugs and can increase the presence of cisplatin in cancer cells, but not to the same extent as copper complexes [32]. On the other hand, incomplete angiogenesis in cancerous tumors creates large pores, making it more likely for compounds to penetrate them [33]. Therefore, the absence of these two factors in normal cells can be decisive in preventing the entry of the Cu/Na dipicolinic acid complex into normal cells and reducing side effects compared to cisplatin. For this purpose, the cytotoxic properties of the complex and cisplatin were investigated on MRC-5 fetal lung fibroblast cells to test the proposed hypothesis.

As expected, after 72 hours of exposure to the Cu/Na dipicolinic acid complex, even at the highest concentration (100 µM), only 37% of normal cells are destroyed, and none of the concentrations show any adverse effects on MRC-5 fetal lung fibroblast cells (Fig. **5A**). In contrast, cisplatin at all concentrations reduces cell viability to below 50%. The significant difference in the performance of the two compounds on normal cells can be observed from the obtained IC_50_ value, as shown in Fig. (**5B**). It appears that the larger size of the Cu/Na dipicolinic acid complex, compared to cisplatin, is effective in reducing side effects. Of course, other factors such as the high solubility of the complex and the low toxicity of copper to the body may also play a role [13].

### 3.3 Investigation of Genes Involved in Apoptosis and Resistance via Real-Time PCR Method

Given the unique mechanism of copper, which includes halting the cell cycle, activating stress pathways, and inhibiting angiogenesis, it is reasonable to expect that copper can inhibit tumor growth and induce cell death within it. Particularly when utilizing copper complexes, positive outcomes have been achieved in tumor treatment. It has been observed that these complexes possess a strong ability to trigger autophagy and apoptosis in tumor cells [34]. The important point is that Cu^2+^ cannot be absorbed by cells; therefore, complexation by suitable ligands can cause better binding and transport of copper *via* CTR1 [35]. When the copper concentration exceeds the threshold set by homeostasis, cytotoxicity can occur. Copper ions induce abnormal accumulation of thioredoxin and trigger apoptosis by directly binding to thioredoxin, a lipidated component of the mitochondrial Tricarboxylic Acid (TCA) cycle pathway. Fortunately, researchers have discovered that cells primarily reliant on mitochondrial respiration are vulnerable to copper ions [36]. Given that cancer cells have high expression of mitochondrial lipoic acid proteins and rely on mitochondrial respiration for survival [37], it is possible that Copper complex could offer a novel approach to cancer treatment.

Given that apoptosis can be induced through two main pathways, the mitochondria (intrinsic pathway) or the activation of death receptors (extrinsic pathway), we chose to investigate the Bak1 and Bclx genes for the intrinsic pathway, and the Caspase-3 gene for the extrinsic pathway. Nevertheless, both pathways ultimately lead to the activation of Caspases, which are the final executors of cell death [38]. After a 24-hour exposure of the complex and cisplatin to A549 lung cancer cells, the Bak1 gene was increased by the Cu/Na dipicolinic acid complex by 1.56 times compared to the control (Fig. **6**), while cisplatin decreased the expression of this gene compared to the control (Table **1**). Unexpectedly, an increase was observed in the expression of the anti-apoptotic gene Bclx by the Cu/Na dipicolinic acid complex, which was elevated by 3.64 times compared to the control. This resulted in a Bak1/Bclx ratio indicating the activation of the mitochondrial pathway of 0.43 for the Cu/Na dipicolinic acid complex and 1.64 for cisplatin, demonstrating that the complex was not successful in inducing the intrinsic pathway of apoptosis.

Another fact is the activation of death receptors or the extrinsic pathway of apoptosis, which is stimulated by both compounds. This indicates that the involvement of CTR1 may be linked to the activation of Caspase-3. Additionally, the Cu/Na dipicolinic acid complex plays a more significant role in activating CTR1, leading to an increase in the expression of the Caspase-3 gene. Wang *et al*. [39] have also demonstrated that the upregulation of CTR1 in the presence of cisplatin can induce apoptosis and enhance the expression of Caspase-3. Therefore, it can be concluded that, out of the two investigated pathways, the extrinsic pathway for the Cu/Na dipicolinic acid complex has been activated, leading to an increased expression of this gene with greater efficacy compared to cisplatin.

It has been proven that CTR1 plays a significant role in cisplatin drug resistance, leading to extensive research in this area [40]. However, the results in Table **1** indicate that cisplatin did not induce resistance in this cell line, as there was no increase in the expression of the AKT1 gene. Since the expression of the anti-apoptotic Bclx gene is directly linked to AKT1 [41], it can be concluded that the dosage of cisplatin and 24-hour exposure to A549 lung cancer cells do not result in drug resistance. In our study, the expression of both Bclx and AKT1 genes was decreased by cisplatin compared to the control (Fig. **6**). In confirmation of this, Liu *et al*. induced resistance in A549 cells by keeping them in medium containing 5 µg/mL cisplatin for at least 6 months [42]. The Cu/Na dipicolinic acid complex caused a slight increase in the expression of the AKT1 gene compared to the control (1.31 to 1), suggesting that the role of CTR1 and copper in transporting the complex is more likely.

The unique redox properties of copper can impact the physiological activity of cells, either benefiting or harming them, depending on the level of accumulation. Excessive copper can lead to cytotoxicity and cell death, while insufficient copper can result in impaired absorption and transport within cells [43]. Numerous clinical reports have linked high copper exposure to an increased risk of Chronic Obstructive Pulmonary Disease (COPD) [44]. Conversely, studies have shown that the GHK-Cu complex in lung tissue affected by pulmonary fibrosis can reduce inflammation by lowering the levels of inflammatory cytokines, such as TNF-α [3]. As a result, we chose to expose normal lung tissue to the Cu/Na dipicolinic acid complex for 24 hours and analyze the expression of the TNF-α gene, which is related to inflammation, using real-time PCR. As shown in Fig. (**7**), the Cu/Na dipicolinic acid complex significantly reduced TNF-α gene expression, confirming that the dose used will not induce inflammation in normal cells. Additionally, cisplatin did not cause any inflammation or increase gene expression. Therefore, it can be concluded that the complex at a high dose (247 µM) does not trigger inflammation in MRC-5 normal cells, and in fact, leads to a decrease in TNF-α gene expression. This suggests that the Cu/Na dipicolinic acid complex at a low dose may induce apoptosis in cancer cells without causing inflammatory effects in normal cells.

### 3.4 Evaluation of Oxidative Damage by Determining the Amount of MDA

The positive effect of copper on erythrocytes could be a significant advantage in ensuring the safety of the synthesized complex once it enters the circulatory system. Data shows that copper intake up to 7 mg/day does not have pro-oxidant activity and may even protect erythrocytes from oxidation [45]. Copper is also involved in various physiological processes, including angiogenesis, regulation of gene expression, brain development, and immune function [46]. Additionally, defense against oxidative damage relies heavily on copper-containing superoxide dismutase [47]. Therefore, it is necessary to incubate erythrocytes with the Cu/Na dipicolinic acid complex to assess the level of oxidative stress it may cause. The typical method for assessing oxidative stress involves measuring the amount of MDA produced through lipid peroxidation of unsaturated fatty acids. To conduct this evaluation, the Cu/Na dipicolinic acid complex and cisplatin were exposed to erythrocytes for a 24-hour period. The results depicted in Fig. (**8**) indicate that cisplatin led to an increase in MDA levels, whereas the complex, at both concentrations tested, did not alter MDA levels.

Therefore, based on this straightforward test, we can cautiously infer that the Cu/Na dipicolinic acid complex, unlike cisplatin, may not have a detrimental impact on blood cells within the circulatory system. However, it is important to note that further research is necessary before drawing definitive conclusions.

## CONCLUSION

In summary, our findings highlight the potential of the Cu/Na dipicolinic acid complex as a promising alternative to cisplatin for the treatment of lung cancer. By utilizing the CTR1 pathway for cellular entry, this complex demonstrated enhanced cytotoxicity against A549 cancer cells while exhibiting no harmful effects on normal MRC-5 fetal lung fibroblasts. Unlike cisplatin, which causes significant oxidative stress in erythrocytes and cytotoxicity in normal cells even at low doses, the Cu/Na dipicolinic acid complex exhibited excellent biocompatibility. It does not induce inflammatory responses or oxidative stress, even at high doses. Furthermore, its mechanism of apoptosis induction *via* the extrinsic pathway, accompanied by an increased Caspase-3 gene expression, parallels cisplatin while offering a safer profile. These advantages position the Cu/Na dipicolinic acid complex as a potential chemotherapeutic agent with reduced side effects, paving the way for more effective and less harmful cancer treatments.

## AUTHORS' CONTRIBUTIONS

The authors confirm their contribution to the paper as follows: conceptualization: DG; methodology: ZR; data curation: AH; validation: MHM; draft manuscript: CL.

All authors reviewed the results and approved the final version of the manuscript.

## Figures and Tables

**Fig. (1):**
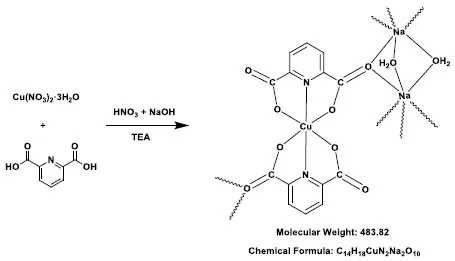
Shows a schematic image of the Cu/Na dipicolinic acid complex. The formula weight of one unit of this polymer complex is 483.82 g/mol, and its formula is C_14_H_18_CuN_2_Na_2_O_10_.

**Fig. (2):**
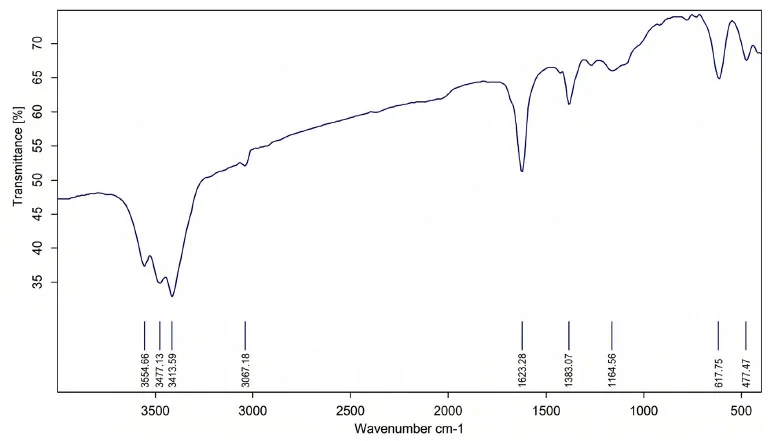
Shows the FTIR spectrum of Cu/Na dipicolinic acid complex obtained after mixing with KBr.

**Fig. (3):**
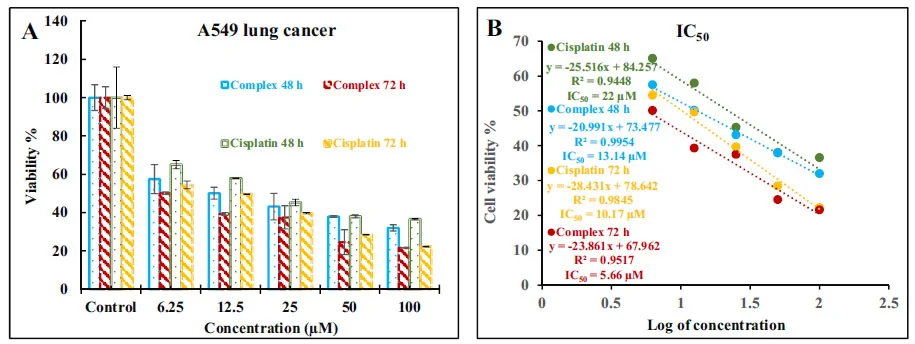
Part A, displays the percentage of lung cancer cell death achieved through the MTT assay. In part B, IC_50_ values are calculated after 48 and 72 hours of exposure to the complex and cisplatin. The IC_50_ values are determined from the equation of the line drawn based on the logarithm of the concentration relative to the percentage of cell viability.

**Fig. (4):**
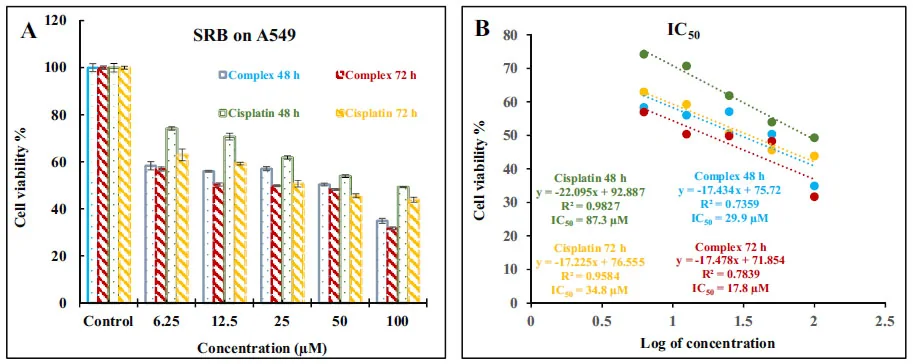
Part **A**, displays the percentage of lung cancer cell death achieved by measuring the protein content of the cells. In part **B**, IC_50_ values are calculated after 48 and 72 hours of exposure to the complex and cisplatin.

**Fig. (5):**
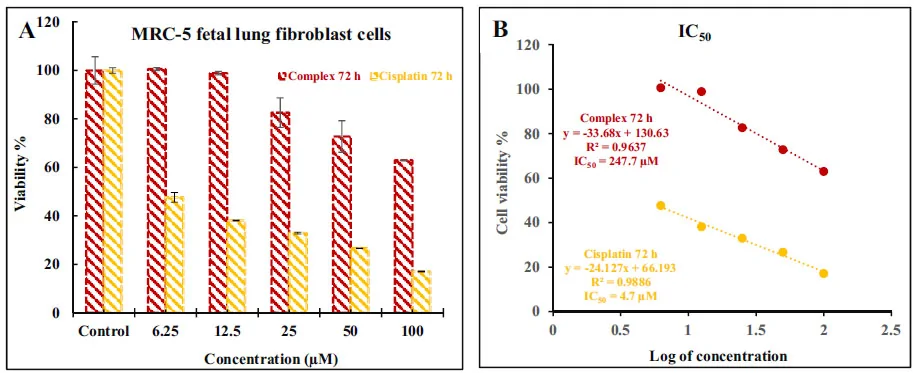
Part A, displays the cytotoxic effects of the complex and cisplatin on normal cells. Part B shows the IC_50_ values obtained from the 72-hour treatment.

**Fig. (6):**
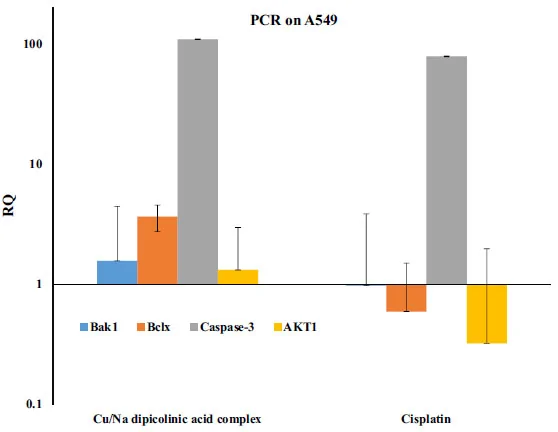
Shows the expression level of genes involved in apoptosis and resistance of cancer cells when treated with complex and cisplatin.

**Fig. (7):**
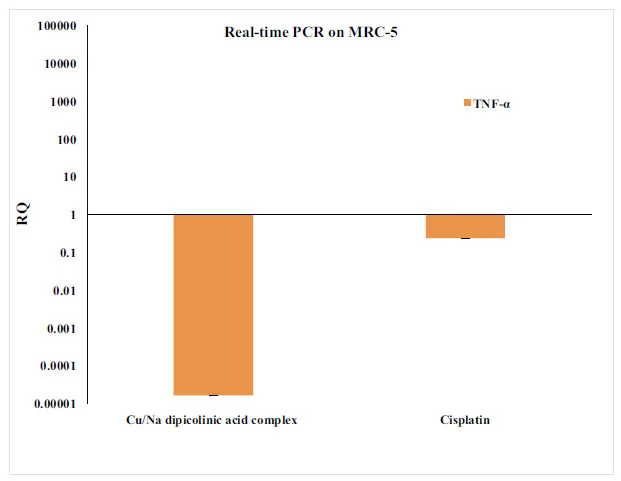
Shows the level of expression of the TNF-α gene when the complex and cisplatin were adjacent to normal cells.

**Fig. (8):**
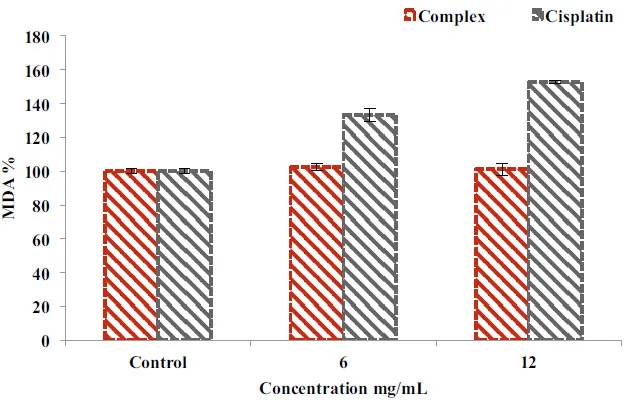
Presents the levels of MDA produced by erythrocytes after exposure to Cu/Na dipicolinic acid complex and cisplatin.

**Table 1:** Gene, sequence, RQ value, and ΔΔCT.

**Samples**	**Cell line**	**Genes and Sequences**	**ΔΔCT**	**RQ**
Cu/Na dipicolinic acid complex	A549 lung cancer cells	Bak1 F: TCTGGGACCTCCTTAGCCCT R: AATGGGCTCTCACAAGGGTATT	-0.64	1.56
Cisplatin			0.05	0.97
Control			0	1
Cu/Na dipicolinic acid complex		Bclx F: TGGAAAGCGTAGACAAGGAGA R: TGCTGCATTGTTCCCATAGA	-1.865	3.64
Cisplatin			0.76	0.59
Control			0	1
Cu/Na dipicolinic acid complex		Caspase-3 F: TGCAGTCATTATGAGAGGCAAT R: AAGGTTTGAGCCTTTGACCA	-6.775	109.5
Cisplatin			-6.3	78.8
Control			0	1
Cu/Na dipicolinic acid complex		AKT1 F: CCAACACCTTCATCATCCGC R: AGTCCATCTCCTCCTCCTCC	-0.395	1.31
Cisplatin			1.665	0.32
Control			0	1
Cu/Na dipicolinic acid complex	MRC-5 fetal lung fibroblast cells	TNF-α F: CCAGAGGAAGAAGCAACACA R: GGAATGAATGCCCACTCCTT	15.925	0.000016
Cisplatin			2.115	0.23
Control			0	1

## Data Availability

All data and findings utilized in this study will be provided by the corresponding author upon reasonable request.

## LIST OF ABBREVIATIONS

Cu/Na: Copper/sodium
MTT assay: Colorimetric Assay used a Yellow Tetrazolium Salt
SRB assay: Colorimetric Method used Sulforhodamine B
MRC-5: Medical Research Council Cell Strain 5
AKT1: AKT Serine/Threonine Kinase 1
TNF-α: Tumor Necrosis Factor alpha
PCR: Polymerase Chain Reaction
DNA: Deoxyribonucleic Acid
Bclx: B-cell lymphoma-extra
Bak1: BCL2 Antagonist/Killer 1
DMSO: Dimethyl Sulfoxide
ELISA: Enzyme-Linked Immunosorbent Assay
